# Prognostic value of plasma DPP4 activity in ST-elevation myocardial infarction

**DOI:** 10.1186/s12933-017-0553-3

**Published:** 2017-06-06

**Authors:** Jing-Wei Li, Yun-Dai Chen, Wei-Ren Chen, Qi You, Bo Li, Hao Zhou, Ying Zhang, Tian-Wen Han

**Affiliations:** 1 Department of Cardiology, People’s Liberation Army General Hospital, No. 28 Fuxing Road, Wukesong, Haidian District, Beijing, 100853 China; 2 Department of Cardiology, Xinqiao Hospital, Third Military Medical University, Chongqing, China

**Keywords:** Dipeptidyl peptidase 4, ST elevation myocardial infarction, Cardiovascular system, Biomarkers, Prognosis

## Abstract

**Background:**

Dipeptidyl peptidase-4 (DPP4) regulates blood glucose levels and inflammation, and it is also implicated in the pathophysiological process of myocardial infarction (MI). Plasma DPP4 activity (DPP4a) may provide prognostic information regarding outcomes for ST-segment elevation MI (STEMI) patients.

**Methods:**

Blood samples were obtained from 625 consecutively admitted, percutaneous coronary intervention-treated STEMI patients with a mean age of 57 years old. DPP4a was quantified using enzymatic assays.

**Results:**

The median follow-up period was 30 months. Multivariate Cox-regression analyses (adjusted for confounding variables) showed that a 1 U/L increase of DPP4a did not associate with risks of major adverse cardiac or cerebrovascular events (MACCE), cardiovascular mortality, MI, heart failure readmission, stroke, non-cardiovascular mortality and repeated revascularization. However, in a subset of 149 diabetic STEMI patients, DPP4a associated with an increased risk of MACCE (HR 1.16; 95% CI 1.04–1.30; *p* = 0.01).

**Conclusions:**

DPP4a did not associate with cardiovascular events and non-cardiovascular mortality in non-diabetic STEMI patients. However, DPP4a may be associated with future MACCE in diabetic STEMI patients.

*Trial registration* NCT03046576, registered on 5 February, 2017, retrospectively registered

**Electronic supplementary material:**

The online version of this article (doi:10.1186/s12933-017-0553-3) contains supplementary material, which is available to authorized users.

## Background

Dipeptidyl peptidase 4 (DPP4) was identified as a cell surface protein that cleaved amino-terminal dipeptides containing either l-proline or l-alanine at the second to last position. DPP4 inactivated glucagon-like peptide-1 (GLP-1), a member of the incretin metabolic hormone family, thus involving it in glucose metabolism [1]. Widespread expression of DPP4 on the surface of myocardial and endothelial cells, and the non-enzymatic function of it as a signaling and binding protein suggest a function in cardiovascular regulation [2]. Data elucidating a potential role for DPP4 in heart failure (HF) have conflicted. The DPP4 inhibitor saxagliptin led to a 27% increase in hospitalization for HF in diabetic patients who had a history of, or were at risk for, cardiovascular events [3]. Use of a DPP4 inhibitor associated with reduced risk of hospitalization for HF when compared with patients receiving antidiabetic sulphonylurea drugs [4]. A recent meta-analysis showed that the relative effects of DPP4 inhibitors on HF risk were uncertain [5]. Conversely, the role of DPP4 in myocardial infarction (MI) was largely consistent across studies. DPP4 inhibition during MI events played a protective role. Either genetic disruption or chemical inhibition of DPP4 improved functional recovery after ischemia/reperfusion injury in an animal MI model [6], and DPP4 inhibitors improved left ventricular diastolic function in diabetic patients with acute MI [7].

DPP4 also exists in a soluble form in the plasma, where it is thought to be shed from the membranes of endothelial cells, while maintaining enzymatic activity [8]. Increased plasma DPP4 activity (DPP4a) predicted both sub-clinical and new-onset atherosclerotic events [9, 10]. Additionally, DPP4a correlated with adverse cardiovascular outcomes in HF [11] as well as sub-clinical left ventricular dysfunction in diabetic patients [12]. Our previous work found that DPP4a was significantly lower in MI patients compared with patients experiencing only chest pain or unstable angina, and DPP4a associated with inpatient no-reflow and major bleeding events in STEMI patients [13]. The no-reflow phenomenon [14] and major bleeding events [15] independently associated with worsened in-hospital and long-term prognoses. Thus, it was hypothesized here that DPP4a may be associated with adverse cardiovascular events during the long-term follow-up period in these patients.

## Methods

### Study population

The People’s Liberation Army General Hospital (PLAGH) is a tertiary referral center in Beijing, China. A total of 841 STEMI patients were consecutively admitted between January 2013 and September 2015 to the department of cardiology, PLAGH. All of the 803 STEMI patients who agreed to participate gave written informed consent. The study protocol was reviewed and approved by the ethics committee of the PLAGH and the Beijing Ethics Association, and is in accordance with the Declaration of Helsinki.

Blood samples were collected on the first morning after admission for all patients with acute myocardial infarction. Plasma samples were frozen at −80 °C until further analyses. Percutaneous coronary intervention (PCI) treatments were administered to 712 participants. Major exclusion criteria for PCI included: conditions requiring treatment with coronary artery bypass grafting (CABG) or thrombolytic therapy according to the current standard guidelines [16], uncontrolled hypertension, any substantial trauma, or increased risk of severe bleeding. To enhance homogeneity of the sample and to ensure examination of a representative cohort in the context of contemporary treatment modalities, we further excluded patients with cancer (n = 24; cancer can affect both DPP4a and the outcomes of interest [17]), and patients who were taking a DPP4 inhibitor (n = 26) or a GLP-1 analogue (n = 16). Patients were followed for a median of 30 months (interquartile range 23–44 months), and 21 patients were lost to follow-up. Thus, the final sample size was 625 patients.

The age range of patients was 28–88 years old. Participants did not experience cardiogenic shock, and survived for at least 24 h after PCI treatment. STEMI events were defined as MI with typical cardiac ischemic symptoms, as well as electrocardiographic ischemic changes with ST-segment elevations of >0.1 mm in at least two contiguous leads, and positive cardiac troponin tests. PCI procedures were performed according to current AHA PCI standard guidelines [18]. Post-procedural medications were given according to ACC/AHA guidelines and included: aspirin, clopidogrel, β-blockers, angiotensin-converting enzyme inhibitors, angiotensin receptor blockers, calcium channel blockers, nitrates, antidiabetic agents and statins [19].

### Outcome events and follow-up

All clinical and demographic properties of the patients were recorded from hospital files and computer records. The primary endpoint for this study was a major adverse cardiac or cerebrovascular event (MACCE), defined as either: cardiovascular (CV) death, non-fatal myocardial infarction, heart failure, or stroke. Other endpoints of interest included non-CV death and repeated revascularization. Outcome status, date and etiologies were obtained from follow-up outpatient visits, inpatient clinical records of re-admitted patients, or by telephone interviews in which the moderators were blinded to patients’ DPP4a. There was no adjudication. Revascularization was defined as repeated PCI or bypass grafting of either infarct-related arteries or non-infarct-related arteries, occurring due to either ischemic symptoms (stable/unstable angina or re-infarction) or detections of ischemic events by non-invasive tests. Stroke was defined using World Health Organization-defined criteria [20]. Hypertension was defined as either a blood pressure level of ≥140/90 mmHg by three separate resting sphygmomanometer measurements or the use of an anti-hypertensive agent. Throughout this article, any reference to plasma glucose concentrations pertained to measurements obtained after an overnight fast of at least 8 h, occurring within 24 h of admission. Patients were considered to have type-2 diabetes mellitus (T2DM) if they were previously diagnosed or used anti-diabetic agents prior to admission.

### Biochemical measurements

All biomarker measurements were performed by investigators who were blinded to patients’ characteristics and outcomes. Fast plasma glucose, total cholesterol, low-density lipoprotein (LDL) cholesterol, high-density lipoprotein (HDL) cholesterol, triglycerides, N-terminal pro B-type natriuretic peptide (NT-proBNP), creatinine, creatine kinase-MB (CK-MB), alanine aminotransferase (ALT), aspartate aminotransferase (AST) and gamma-glutamyltransferase (GGT) levels were measured using automated enzymatic methods (Cobas^®^, Roche, Germany). DPP4 activities were determined in EDTA-treated plasma samples by measuring rates of p-nitroaniline (pNa) cleavage from the synthetic substrate H-Gly-Pro-pNa (L1880, Bachem, Switzerland) by DPP4, as previously described [13]. Briefly, 5 µL plasma samples were added to 150 µL of 50 mM tris–HCl containing 1 mM H-Gly-Pro-pNa. DPP4 activity were expressed as the amount of pNa cleaved per minute per liter (U/L).

### Statistical analyses

Data distributions were evaluated for normality by plotting both probability plot and quantile–quantile plot. Continuous variables were expressed as means (±SD), or medians (interquartile ranges) depending on normality. Categorical variables were expressed as counts (percentages). ANOVA, Kruskal–Wallis and Chi square tests were used to compare normally distributed variables, and non-normally distributed continuous and discrete variables, respectively. To evaluate the prognostic value of DPP4a, patients were stratified into DPP4a tertiles. Survival analyses were performed with Kaplan–Meier methods with stratifications by DDP4a tertiles, and results were statistically evaluated with log-rank tests. Cox proportional hazard regression models were applied to query the data for independent predictors of outcomes. Participants were censored upon first presentation of the combined endpoint. To select variables for inclusion in multivariate analyses, univariate Cox proportional hazards models using forward variable selection methods were used. Variables were considered significant in the univariate models when *p* < 0.05. Other factors included in the multivariate models have been previously shown to associate with MACCE [21]. Model 1 adjusted for age and gender. Model 2, the fully adjusted model, also adjusted for: body mass index; levels of: creatinine, triglycerides, aspartate aminotransferases, CK-MB, pro-brain natriuretic peptides, and fasting plasma glucose; hypertension; smoking; previous myocardial infarction; and use of: ACEI, ARB, statins, beta-blockers, calcium channel blockers or diuretics. All statistical tests were two-tailed and differences were considered significance if *p* values were less than 0.05. All analyses were performed with SPSS 13.0 software (SPSS, IL, USA).

## Results

Demographics of the included and excluded patient populations are given in Additional file 1: Table S1. No significant differences between characteristics of the two groups were observed. Demographics of the included participant populations are given in Table 1. The cohort comprised 519 men (81.9%) and 106 women (19.1%) with a mean age of 57.4 ± 11.4 years. The median DPP4a was 27.49 ± 8.76 U/L. No difference in activity level was found between genders (male: 27.49 ± 8.78 U/L, female: 27.46 ± 8.72 U/L; *p* = 0.97), or between diabetic and non-diabetic patients (diabetic: 26.38 ± 9.21 U/L, non-diabetic: 27.83 ± 8.61 U/L; *p* = 0.08). Increased DPP4a (by tertiles) negatively associated with histories of hypertension. Conversely, DPP4a positively associated with current smoking status, and levels of ALT and AST (Table 1).

**Table 1:** Patients’ characteristics, stratified by DPP4a tertiles

	Total	T1	T2	T3	p value
U/L		<23.45	23.45–31.30	>31.30	
N	625	207	210	208	–
Age (years)	57.4 ± 11.4	58.1 ± 11.7	58.2 ± 11.5	55.8 ± 11.0	0.049
Male, n (%)	519 (81.9)	174 (84.1)	172 (81.9)	173 (83.2)	0.35
BMI	25.8 ± 3.4	25.8 ± 3.6	25.7 ± 3.3	26.0 ± 3.3	0.61
Hypertension, n (%)	306 (50.9)	117 (56.3)	103 (49.0)	86 (41.3)	0.01
Type 2 diabetes, n (%)	149 (23.8)	58 (28.0)	49 (23.3)	42 (20.2)	0.17
Current smoker, n (%)	282 (45.1)	80 (38.6)	94 (44.7)	108 (51.9)	0.03
Ex-smoker, n (%)	75 (12.0)	31 (15.0)	25 (11.9)	19 (9.1)	0.19
Previous MI, n (%)	77 (12.3)	23 (11.1)	23 (11.0)	31 (14.9)	0.39
Anterior MI, n (%)	322 (51.5)	111 (53.6)	110 (52.6)	101 (48.7)	0.56
Medications, n (%)					
Aspirin	624 (99.8)	207 (100.0)	209 (99.5)	208 (100)	0.37
ACEI/ARB	579 (93.0)	195 (94.2)	190 (90.5)	194 (93.3)	0.32
β-Blocker	554 (88.6)	189 (91.3)	182 (86.7)	183 (88.0)	0.36
Clopidogrel	610 (97.6)	204 (98.6)	203 (97.1)	203 (97.6)	0.45
Diuretics	271 (43.4)	98 (47.3)	94 (44.8)	79 (38.0)	0.14
Calcium channel blocker	114 (18.2)	44 (21.3)	41 (19.5)	29 (13.9)	0.13
Statin	623 (99.7)	206 (99.5)	209 (99.5)	208 (100)	0.61
Nitrate	559 (89.4)	191 (92.3)	183 (87.1)	185 (88.9)	0.22
Total cholesterol (mmol/L)	4.10 ± 1.04	4.03 ± 1.01	4.00 ± 1.00	4.27 ± 1.08	0.02
Triglyceride (mmol/L)	1.57 ± 0.85	1.54 ± 0.81	1.53 ± 0.80	1.64 ± 0.93	0.34
HDL cholesterol (mmol/L)	1.04 ± 0.29	1.02 ± 0.29	1.01 ± 0.25	1.10 ± 0.31	<0.01
LDL cholesterol (mmol/L)	2.53 ± 0.89	2.47 ± 0.89	2.46 ± 0.85	2.64 ± 0.90	0.08
FPG (mmol/L)	6.06 (5.04–7.79)	6.02 (5.07–8.05)	5.99 (5.01–8.15)	6.19 (5.03–7.78)	0.88
CK-MB (ng/mL)	6.42 (1.82–126.10)	4.73 (1.81–70.05)	4.71 (1.75–139.20)	12.01 (1.89–202.45)	0.05
cTNT (ng/mL)	0.65 (0.05–4.13)	0.56 (0.05–3.00)	0.54 (0.04–4.16)	1.03 (0.04–5.21)	0.38
Myoglobin (ng/mL)	45.80 (28.47–340.30)	42.89 (28.45–191.68)	39.95 (27.44–303.43)	85.30 (28.58–758.50)	0.03
NT-proBNP (pg/mL)	974 (342–2234)	990 (290–2608)	1139 (383–2241)	773 (337–1921)	0.30
Creatinine (umol/L)	78.50 (68.40–90.40)	79.00 (65.20–91.90)	79.90 (70.15–93.45)	77.30 (68.70–87.20)	0.19
GGT (U/L)	32.60 (21.65–54.15)	30.40 (21.20–47.25)	30.40 (21.50–51.50)	37.90 (23.60–72.30)	<0.01
ALT (U/L)	34.00 (21.10–55.00)	30.40 (19.20–45.30)	30.60 (20.70–54.40)	42.45 (25.45–67.93)	<0.01
AST (U/L)	29.20 (18.80–65.60)	26.65 (17.65–51.98)	29.80 (19.00–68.00)	32.65 (20.55–84.63)	<0.01
Follow-up events, n (%)					
MACCE	47 (7.5)	17 (8.2)	15 (7.1)	15 (7.2)	0.90
CV mortality	24 (3.8)	9 (4.3)	8 (3.8)	7 (3.4)	0.87
Non-CV mortality	9 (1.4)	1 (0.5)	2 (1.0)	6 (2.9)	0.09
Non-fetal MI	9 (1.4)	3 (1.4)	2 (1.0)	4 (1.9)	0.71
HF readmission	12 (1.9)	2 (1.0)	5 (2.4)	5 (2.4)	0.47
Stroke	8 (1.3)	3 (1.4)	3 (1.4)	2 (1.0)	0.88
Repeated revascularization	41 (6.6)	13 (6.3)	10 (4.8)	18 (8.7)	0.27

Data are presented as mean ± SD, numbers (percentages) or median (interquartile range)

*ACEI* angiotensin converting enzyme inhibitors, *ALT* alanine aminotransferase, *ARB* angiotensin receptor blocker, *AST* aspartate aminotransferase, *BMI* body mass index, *NT-proBNP* N-terminal pro B-type natriuretic peptide, *CV* cardiovascular, *CK-MB* MB isoenzyme of creatine kinase, *cTNT* cardiac troponin T, *DPP4a* plasma dipeptidyl peptidase-4 activity, *FPG* fasting plasma glucose, *GGT* g-glutamyl transferase, *HF* heart failure, *HDL* high-density lipoprotein, *LDH* lactate dehydrogenase, *LDL* low-density lipoprotein, *MACCE* major adverse cardiac or cerebrovascular events, *MI* myocardial infarction

**Fig. 1:**
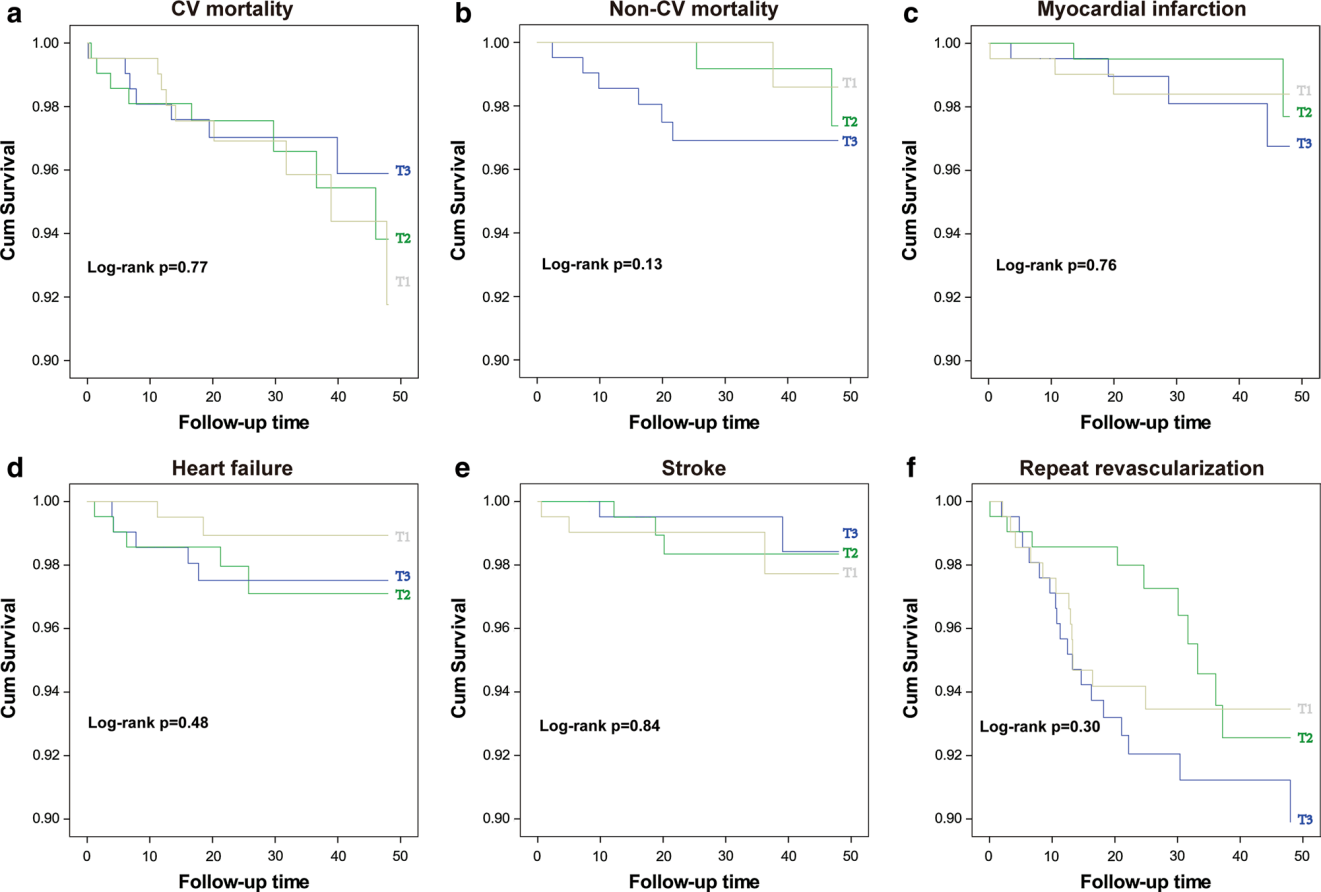
Results of Kaplan–Meier analysis of cumulative event-free rates in STEMI patients, stratified by DPP4a tertiles. As noted, rates of CV death (**a**), non-CV death (**b**), myocardial infarction (**c**), heart failure (**d**), and stroke (**e**) as well as repeated revascularization free-rate (**f**) were not significantly different among DPP4a tertiles

Kaplan–Meier longitudinal analyses showed that patients in the highest DPP4a tertile (DPP4a >31.30 U/L) had similar CV event rates and non-CV mortality rates when compared with patients in the lowest and the middle DPP4a tertiles (all log-rank tests *p* > 0.05; Fig. 1). Multivariate Cox proportional hazards models were used to quantify the adjusted risk of MACCE. In both unadjusted and adjusted models, DPP4a did not associate with: MACCE (adjusted HR [aHR]: 1.01, 95% CI [0.98–1.05] for 1 U/L increase of DPP4a, *p* = 0.53), CV mortality, myocardial infarction, HF readmission, stroke and repeated revascularization. In the fully-adjusted model, DPP4a did not associate with increased incidence of non-CV mortality. An additional sub-group analysis was performed to determine whether increased DPP4a was predictive of MACCE in diabetic or non-diabetic patients. One U/L increase of DPP4a associated with an increasing risk of MACCE in diabetic patients (aHR 1.16 [1.04–1.30], p = 0.01), but not non-diabetic patients (aHR 0.98 [0.94–1.03], p = 0.48) in the fully-adjusted models (Table 2).

**Table 2:** Multivariable cox proportional hazards models for predicting outcomes per 1 U/L increase of DPP4a

	Unadjusted		Model 1		Model 2	
	HR (95% CI)	p value	HR (95% CI)	p value	HR (95% CI)	p
MACCE	1.00 (0.97–1.04)	0.93	1.02 (0.98–1.05)	0.33	1.01 (0.98–1.05)	0.53
CV mortality	1.01 (0.96–1.06)	0.71	1.03 (0.98–1.08)	0.26	1.01 (0.95–1.06)	0.83
Myocardial infarction	1.03 (0.95–1.10)	0.51	1.03 (0.96–1.11)	0.41	1.05 (0.96–1.15)	0.29
HF readmission	1.03 (0.97–1.09)	0.41	1.04 (0.98–1.11)	0.23	1.03 (0.96–1.11)	0.41
Stroke	0.97 (0.89–1.06)	0.52	0.99 (0.91–1.08)	0.78	0.99 (0.89–1.09)	0.78
Non-CV mortality	1.05 (0.98–1.12)	0.18	1.08 (1.00–1.16)	0.04	1.06 (0.97–1.16)	0.18
Repeated revascularization	1.02 (0.99–1.06)	0.22	1.02 (0.99–1.06)	0.26	1.02 (0.98–1.06)	0.31
MACCE (in non-T2DM)	0.99 (0.95–1.03)	0.49	1.00 (0.96–1.04)	0.91	0.98 (0.94–1.03)	0.48
MACCE (in T2DM)	1.04 (0.98–1.11)	0.18	1.05 (0.98–1.21)	0.16	1.16 (1.04–1.30)	0.01

Model 1 adjusted for age and gender; Model 2 adjusted for Model 1 plus body mass index, creatinine, triglyceride, aspartate aminotransferase, CK-MB, NT-pro-brain natriuretic peptide, fasting plasma glucose, hypertension, smoking, previous myocardial infarction and use of ACEI, ARB, statin, β-blocker, calcium channel blocker and diuretics

*CI* confidence interval, *CV* cardiovascular, *DPP4a* plasma dipeptidyl peptidase-4 activity, *HF* heart failure, *HR* hazard ratio, *MACCE* major adverse cardiac or cerebrovascular events, *T2DM* type 2 diabetes

## Discussion

This study demonstrates a lack of evidence that DPP4a predicted MACCE in non-diabetic STEMI patients receiving PCI treatment. However, in this sample, DPP4a may be associated with MACCE in diabetic STEMI patients.

Previous studies have reported that DPP4a did not associate with hypertension in diabetic patients [12], but did associate with new-onset hypertensive events [22]. Our study showed that DPP4a inversely associated with hypertension. In a pre-clinical study, pharmacological inhibition of DPP4 that reduced plasma DPP4a results in improved hypertension rates [23, 24]. In accordance with previous data [25–27], the present study found that patients with higher DPP4a had increased liver transaminase and GGT levels. These data support the hypothesis that DPP4 serum enzymatic activity originates from the liver and is linked to insulin resistance [25, 28]. Previous studies reported that hepatitis C viral infections associated with higher DPP4a [29, 30]. In the current study, only one patient was concomitantly infected with hepatitis C virus (based on preoperative immunological results). Thus, the associations observed between DPP4a and liver transaminases and GGT are not related to hepatitis C virus infections in this population. Although a previous study has shown that DPP4a was higher in diabetic populations [12, 31], this trend was not observed in the current study. However, DPP4a is reduced after MI [13], thus diabetic patients may have more severe DPP4a reductions after STEMI than non-diabetic patients.

DPP4a is considered a potential prognostic marker for cardiovascular diseases. Higher plasma DPP4a was associated with worsened cardiovascular outcomes in heart failure patients [11]. Additionally, DPP4a predicted atherosclerotic events in a healthy cohort [10]. Moreover, inhibition of DPP4 gene products, which down-regulated DPP4a [32], decreased mortality after myocardial infarction in diabetic rats [33]. Expression of the DPP4 gene may affect MACCE of STEMI patients in a GLP-1-dependent way. Bioactive GLP-1 was degraded at its N terminus by DPP4 proteins, and inhibition of DPP4 increased expression of active GLP-1 [34]. GLP-1 could protect the heart from ischemic-reperfusion injury. In humans and in animal models, GLP-1 beneficially affected cardiac contractility, blood pressure and cardiac output [35]. GLP-1 improved outcomes after experimentally-induced myocardial infarction [36]. Our group previously found that GLP-1 analog use before PCI associated with improved left ventricular ejection fraction rates in MI patients after a 3-month follow-up [37, 38]. Moreover, DPP4 protein has additional substrates that participate in responses to ischemic heart disease, such as stromal cell-derived factor-1 alpha (SDF1α), neuropeptide Y and substance P [39]. The plasma levels of these substrates associated with adverse events after MI [40–42]. Inhibition of DPP4 attenuated post-MI cardiac dysfunction and adverse remodeling events in rats [43, 44] and mice [6]. An earlier study from our group identified that DPP4a associated with no-reflow and in-hospital major bleeding events in STEMI patients [13]. We therefore hypothesized that DPP4a associated with long-term prognoses in STEMI patients.

The data reported here showed that DPP4a did not associated with long-term MACCE in STEMI patients. Although circulating DPP4a was changed in MI patients, it did not predict long-term prognoses. In fact, use of a DPP4 inhibitor, which regulated DPP4a, failed to decrease the incidence of MACCE in humans. Three large randomized clinical trials (SAVOR TIMI, NCT01107886; EXAMINE, NCT00968708; and TECOS, NCT00790205) demonstrated that DPP4 inhibitors did not decrease MACCE risk in diabetic patients with CV diseases [45]. In a recent meta-analysis, DPP4 inhibitors did not affect mortality and CV events [46]. DPP4 may play distinct roles in different aspects of cardiovascular disease that collectively lead to an overall neutral effect on cardiovascular events in STEMI patients. A recent study reported that increased circulating DPP4 levels positively and independently associated with coronary artery disease, even in the absence of DM [31]. These data conflict with the present study. However the previous study was a cross-sectional design and did not study long-term outcomes. Moreover acute MI and unstable angina pectoris were not distinguished in the previous study, while the current study only included STEMI patients. Given these fundamental differences, it is reasonable that the results differed in the two studies.

Diabetes has been consistently associated with more severe myocardial infarction. Indeed, diabetic patients have shown more than twofold greater incidence of acute coronary syndromes and cardiovascular diseases, two to fourfold greater incidence of cardiovascular disease-related mortality, and larger infarct sizes compared to non-diabetic patients [47]. Moreover, diabetes confers an approximately threefold greater odds of post-STEMI right ventricular dysfunction, leading to a worsened prognosis for diabetic MI patients [48]. Since DPP4 is a therapeutic target for diabetes, most studies on DPP4 are carried out in diabetic patients. A previous study found that circulating DPP4 was elevated in type 2 diabetic patients, regardless of coronary artery disease status [31], which associated with subclinical left ventricular dysfunction [12] and subclinical atherosclerosis [9] in these patients. DPP4 inhibitor treatments associated with lower risks of mortality as well as MI and ischemic stroke in diabetic patients with pre-existing heart failures [49]. Additionally, vildagliptin, a DPP4 inhibitor, increased survival rates of diabetic, but not non-diabetic mice [33], suggesting that DPP4 may have a unique role in diabetes. In the current study a subgroup analysis was performed on DPP4a and MACCE in diabetic and non-diabetic STEMI patients. We found that higher DPP4a associated with MACCE in 149 diabetic STEMI patients after adjustment for confounding factors. Given that this study was not powered for diabetic patients (only about 25% of the population was diabetic), a strong conclusion cannot be drawn. The predictive effects of plasma markers may be affected by co-morbid diabetes [50]. Additionally, in the above described studies DPP4 had a positive prognostic role in diabetic patients, and our results were obtained after correcting for all confounding factors. Thus, these data support that a positive correlation exists between DPP4a and MACCE in diabetic patients. Nonetheless, a future study is required that is powered to answer this question.

## Limitations

Some limitations existed in the current study. First, the diabetic STEMI patient sub-population was small, as the study was not powered to study this sub-group. Second, the study was limited to inclusion of selected patients from just one center. Third, DPP4 activity was measured at a single time point; additional blood specimens were not collected at other times during patients’ hospital stays or follow-up visits. Peri- or post-operative dynamic changes of DPP4a may provide additional contextual information regarding the role of DPP4 in diabetic STEMI patients.

## Conclusions

In summary, the current study showed that circulating DPP4a was not associated with risks of MACCE in non-diabetic STEMI patients. However, DPP4a may have associated with MACCE in diabetic STEMI patients. This possibility will be explored in a future large-sample multi-center trial.

## Additional file

**Additional file 1: Table S1.** The characteristics of STEMI patients between included and excluded patients.

## Abbreviations

CV: cardiovascular
DPP4: dipeptidyl peptidase-4
DPP4a: plasma DPP4 activity
HR: hazard ratio
MACCE: major adverse cardiac or cerebrovascular events
PCI: percutaneous coronary intervention
STEMI: ST-segment elevation myocardial infarction
