# Supplementary material for: Prognostic value of plasma DPP4 activity in ST-elevation myocardial infarction
Source: Cardiovasc Diabetol. 2017 Jun 6;16:72. doi: 10.1186/s12933-017-0553-3 (PMC5461628; doi:10.1186/s12933-017-0553-3)
Supplement: Supplementary file 1 — Additional file 1: Table S1. The characteristics of STEMI patients between included and excluded patients. [file 12933_2017_553_MOESM1_ESM.docx]

Table S1. The characteristics of STEMI patients between included and excluded patients.

|  | Included patients | Excluded patients | P |
| --- | --- | --- | --- |
| N | 625 | 216 | - |
| Age (y) | 57.4±11.4 | 59.1±12.5 | 0.07 |
| Male, n (%) | 519（81.9） | 175（81.0） | 0.50 |
| BMI | 25.8±3.4 | 25.6±3.5 | 0.46 |
| Hypertension, n (%) | 296（50.9） | 106（49.1） | 0.66 |
| Type 2 diabetes, n (%) | 145 (23.8) | 43（19.9） | 0.32 |
| Current smoker, n (%) | 282 (45.1) | 101（46.8） | 0.68 |
| Ex-smoker, n (%) | 75 (12.0) | 23（10.6） | 0.59 |
| Previous MI, n (%) | 77 (12.3) | 34（15.7） | 0.20 |
| Anterior MI, n (%) | 323 (51.6) | 111（53.6） | 0.94 |
| Medications, n (%) |  |  |  |
| Aspirin | 624 (99.8) | 214（99.1） | 0.10 |
| ACEI/ARB | 579 (93.0) | 191（88.4） | 0.17 |
| β-blocker | 554 (88.6) | 184（85.2） | 0.29 |
| Clopidogrel | 610 (97.6) | 211（97.7） | 0.95 |
| Statin | 623 (99.7) | 213（98.6） | 0.08 |
| Nitrate | 559 (89.4) | 198（91.7） | 0.40 |
| Total cholesterol (mmol/L) | 4.10±1.04 | 4.19±1.07 | 0.28 |
| Triglyceride (mmol/L) | 1.57±0.85 | 1.49±0.78 | 0.22 |
| HDL cholesterol (mmol/L) | 1.04±0.29 | 1.08±0.32 | 0.09 |
| LDL cholesterol (mmol/L) | 2.53±0.89 | 2.56±0.92 | 0.67 |
| FPB (mmol/L) | 6.06 (5.04-7.79) | 6.06 (5.22-7.90) | 0.50 |
| CK-MB (ng/mL) | 6.42 (1.82-126.10) | 13.87 (2.13-178.83) | 0.10 |
| cTNT (ng/mL) | 0.65 (0.05-4.13) | 0.71 (0.04-0.98) | 0.51 |
| Myoglobin (ng/mL) | 45.80 (28.47-340.30) | 130.50 (28.45-191.68) | 0.13 |
| Pro-BNP (pg/mL) | 974 (342-2234) | 1241 (437-2279) | 0.30 |
| Creatinine (umol/L) | 78.50 (68.40-90.40) | 77.70 (64.25-88.80) | 0.23 |
| GGT (U/L) | 32.60 (21.65-54.15) | 33.00 (21.90-50.20) | 0.95 |
| ALT (U/L) | 34.00 (21.10-55.00) | 31.60 (20.90-51.25) | 0.51 |
| AST (U/L) | 29.20 (18.80-65.60) | 28.60 (17.70-62.60) | 0.73 |
| DPP4a (U/L) | 27.49±8.76 | 27.47±8.17 | 0.98 |

Data are presented as mean ± SD, numbers (percentages) or median (interquartile range). ACEI, angiotensin converting enzyme inhibitors; ALT, alanine aminotransferase; ARB, angiotensin receptor blocker; AST, aspartate aminotransferase; BMI, body mass index; BNP, brain natriuretic peptide; CK-MB, MB isoenzyme of creatine kinase; cTNT, cardiac troponin T; DPP4a, plasma dipeptidyl peptidase-4 activity; FPG, fasting plasma glucose; GGT, g-glutamyl transferase; HDL, high-density lipoprotein; LDL, low-density lipoprotein; MI, myocardial infarction.
